# Supplementary material for: Separator with high ionic conductivity enables electrochemical capacitors to line-filter at high power
Source: Nat Commun. 2025 Mar 20;16:2772. doi: 10.1038/s41467-025-58064-2 (PMC11926240; doi:10.1038/s41467-025-58064-2)
Supplement: Supplementary file 1 — Supplementary Information [file 41467_2025_58064_MOESM1_ESM.pdf]

Supplementary Information for

Separator with high ionic conductivity enables electrochemical capacitors to line-filter at high power

Yajie Hu[1], Puying Li[1], Guobin Lai[2,3], Bing Lu[1], Haiyan Wang[1], Huhu Cheng[1], Mingmao Wu[2], Feng Liu[3], Zhi-Min Dang[4], Liangti Qu[1*]

1 Key Laboratory of Organic Optoelectronics & Molecular Engineering, Ministry of Education, Department of Chemistry, Tsinghua University; Beijing, 100084, P. R. China

2 Key Laboratory of Eco-materials Advanced Technology, College of Materials Science and Engineering, Fuzhou University; Fuzhou, Fujian, 350108, P.R. China

3 State Key Laboratory of Nonlinear Mechanics, Institute of Mechanics, Chinese Academy of Sciences; Beijing, 100190, P. R. China

4 State Key Laboratory of Power System Operation and Control, Department of Electrical Engineering, Tsinghua University, Beijing; 100084, P. R. China

*Correspondence: lqu@mail.tsinghua.edu.cn

This file includes:

Supplementary Note 1 (Pages S3-S3)

Supplementary Figures 1-41 (Pages S4-S45)

Supplementary Tables 1-3 (Pages S46-S48)

Supplementary References (Pages S49-S51)

Supplementary Note 1 | Theoretical calculation of the phase angle and capacitance varying with separator thicknesses.

The electrochemical line-filtering capacitor can be equivalent to a simple resistor-capacitor circuit (Supplementary Fig. 41). The separator's ionic resistance is assigned to R_s. Given the transmission-line-like behavior of electrode materials, we use a constant phase angle element (CPE_m) for its simulation. R_L represents the leakage coming from the soft short-circuit caused by separator breakage.

The calculation is conducted by ZView2 software. The calculation assumes that the separator's porosity, density, and electrolyte content remain the same. Based on the statistical results gathered from the literature (Table S2), we use the parameters as follows for calculation:

CPE_m-T (capacitance) = 1 mF cm^{-2}, CPE_m-P (phase angle component) = 0.922 (the corresponding R_m is 0.29 Ω cm^2), $R_L = 1\times10^6$ Ω cm^2, R_s = 1.075 Ω cm^2, 0.1075 Ω cm^2 or 0.01075 Ω cm^2 for the separator thickness of 100 μm, 10 μm or 1 μm, respectively.

Based on the calculation results, for the separator thickness of 100 μm, 10 μm, and 1 μm, the phase angle at 120 Hz shows a decline from −59.9° to −80.4°, and to −82.8°; and the volumetric capacitance changes from 38 mF cm^{-2} to 256 mF cm^{-2}, and to 464 mF cm^{-2}, respectively.

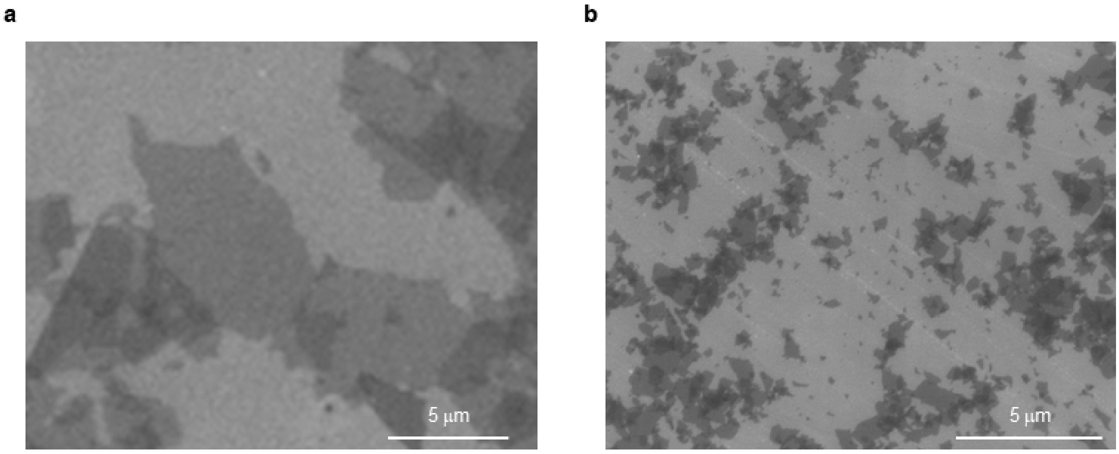

Supplementary Fig. 1 | Sheet size reduction of GO sheets. a, SEM image of original GO sheets. **b**, SEM image of GO nanosheets after 30 mins' ultrasonication.

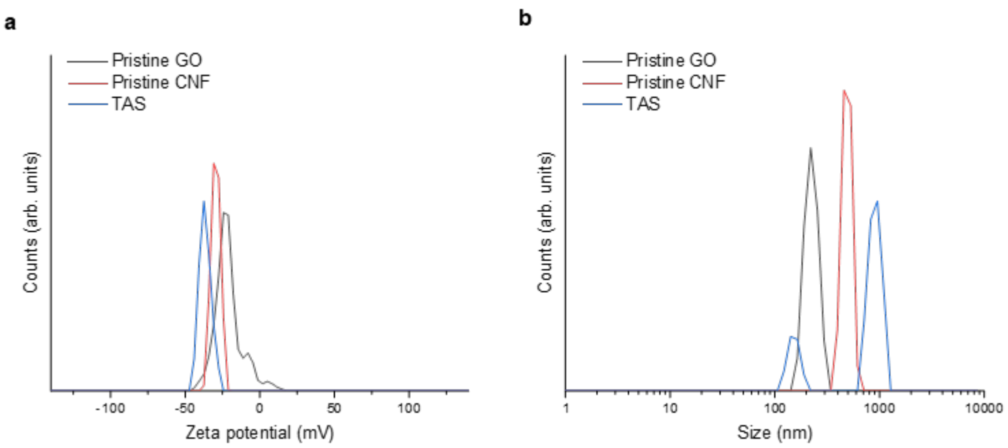

Supplementary Fig. 2 | Tracing of Zeta potential and size distribution of CNFs and GO sheets during the in-solution assembly process. a, Zeta potentials of pristine GO, pristine CNF, and TAS. **b**, Particle size distribution of pristine GO, pristine CNF, and TAS.

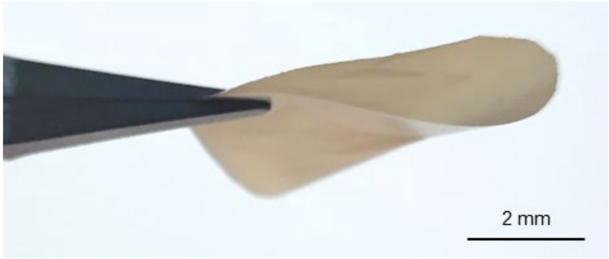

Supplementary Fig. 3 | Optical image of TAS being bent 180°.

a

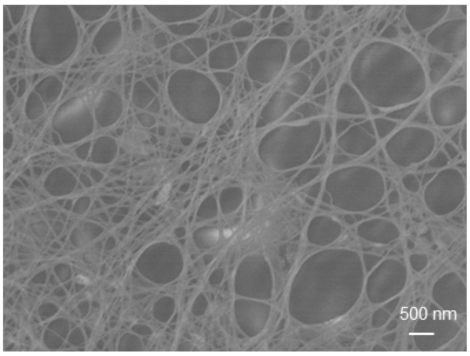

b

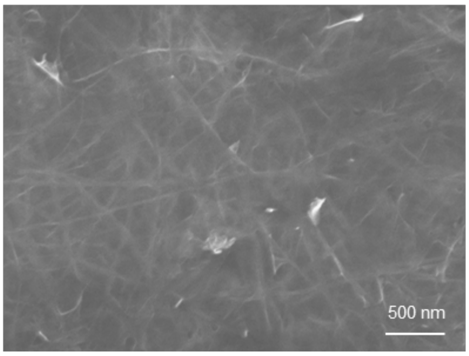

Supplementary Fig. 4 | SEM images of TAS with a lesser thickness to show the thread-anchor structure (a) and TAS with a normal thickness (b).

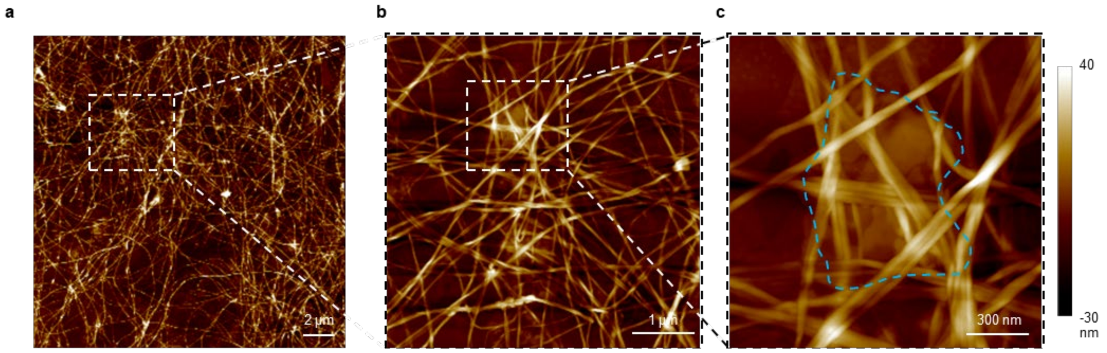

Supplementary Fig. 5 | AFM images of the thread-anchor structure of TAS. a, Low magnification AFM image of TAS showing the thread skeleton with macro pores. **b**, Medium magnification AFM image showing the interconnection between GO anchors and CNF threads. **c**, High magnification AFM image showing that the GO anchor is amidst CNF threads. The turquoise dashed circle marks the GO anchor.

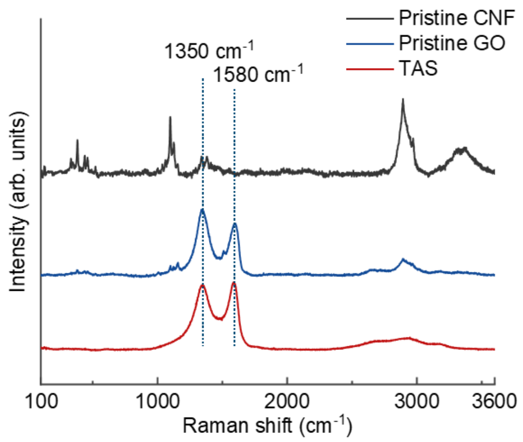

Supplementary Fig. 6 | Raman spectra of pristine CNF, pristine GO, and TAS.

The dashed lines indicate the D band and G band of GO.

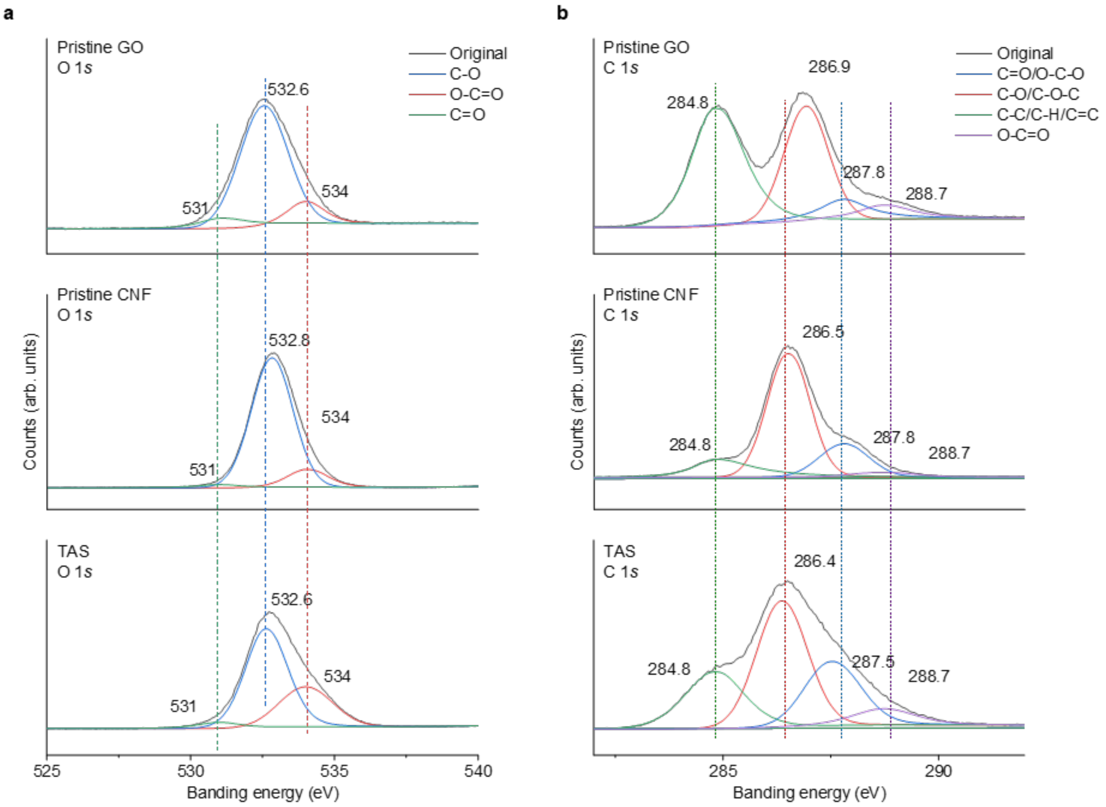

Supplementary Fig. 7 | XPS spectra of pristine GO, pristine CNF, and TAS. a, O 1*s* spectra. **b**, C 1*s* spectra.

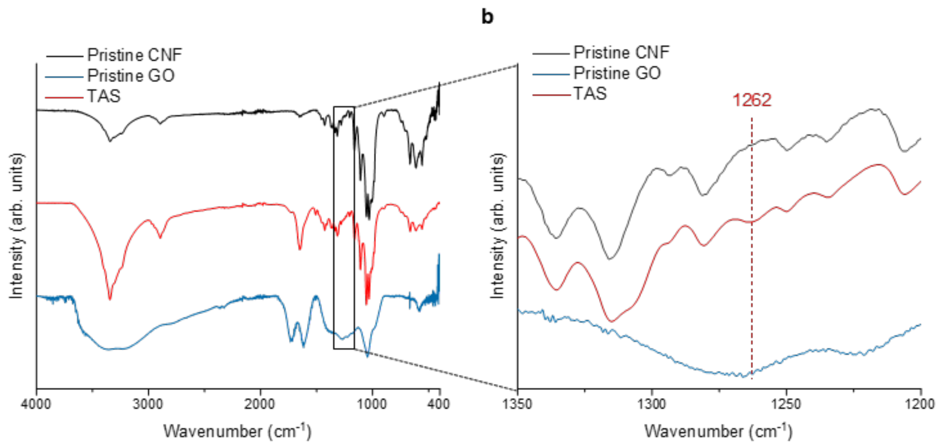

Supplementary Fig. 8 | Infrared spectra of pristine CNF, pristine GO, and TAS. a, The overall spectrum. **b**, The enlarged spectrum showing the newly emerged peak at 1262 cm^{-1}, which corresponds to the C–O–C bond in the ester bond.

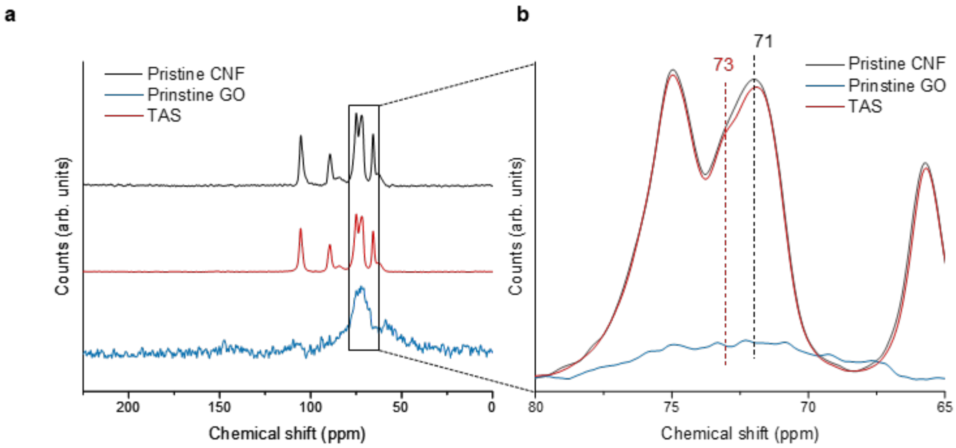

Supplementary Fig. 9 | ^{13}C solid-state nuclear magnetic spectra of pristine CNF, pristine GO, and TAS. a, The overall spectrum. **b**, The enlarged spectrum showing the newly emerged shoulder peak at 73 ppm, which corresponds to a different chemical environment carbon in the C–O bond.

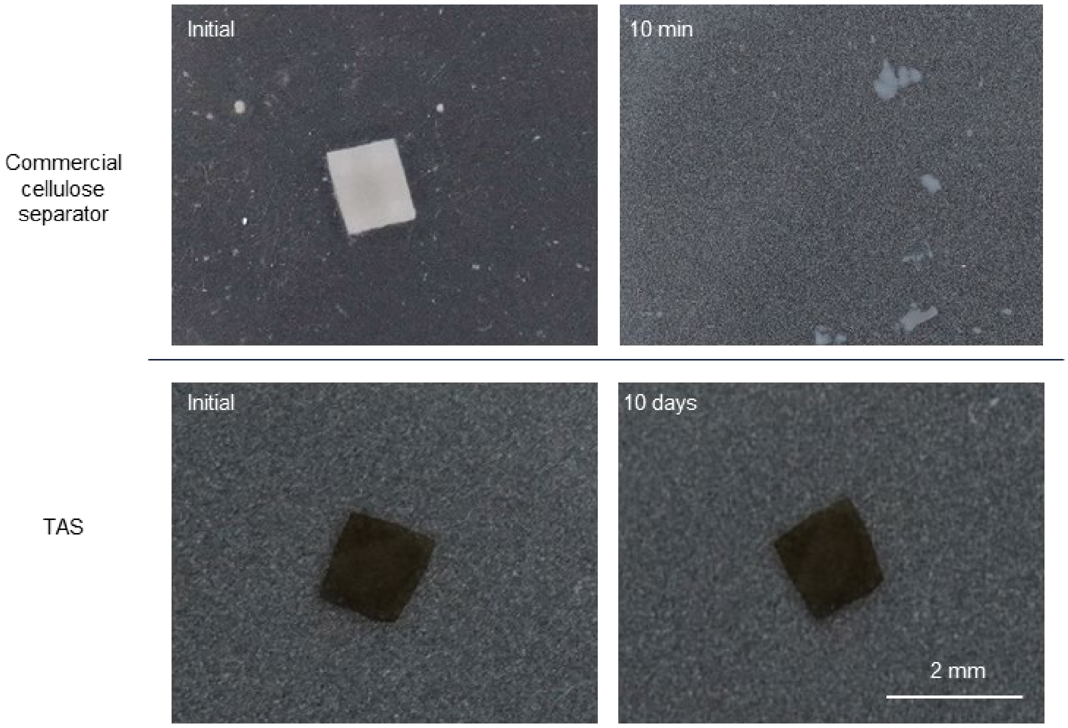

Supplementary Fig. 10 | Chemical stability test of commercial cellulose separator (upper panel) and TAS (bottom panel) in a 5M H_2SO_4 electrolyte.

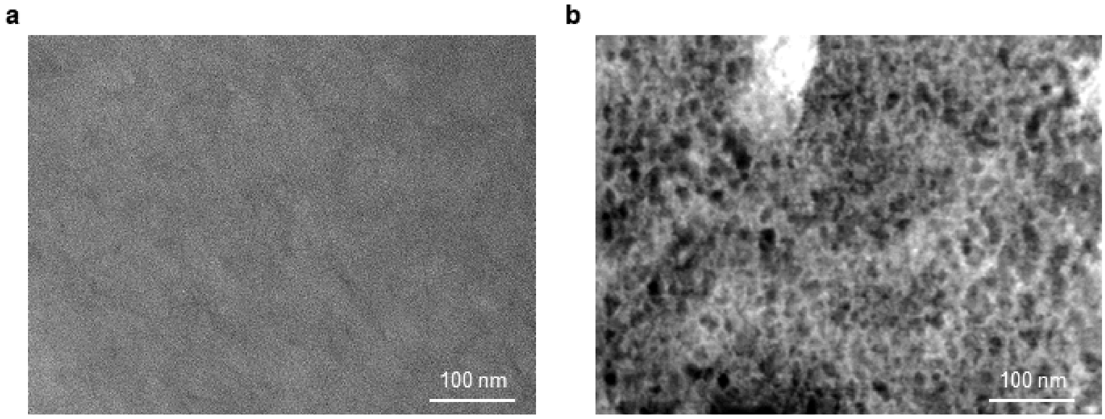

Supplementary Fig. 11 | HAADF-TEM images of PEDOT:PSS, before (a) and after (b) methanol treatment.

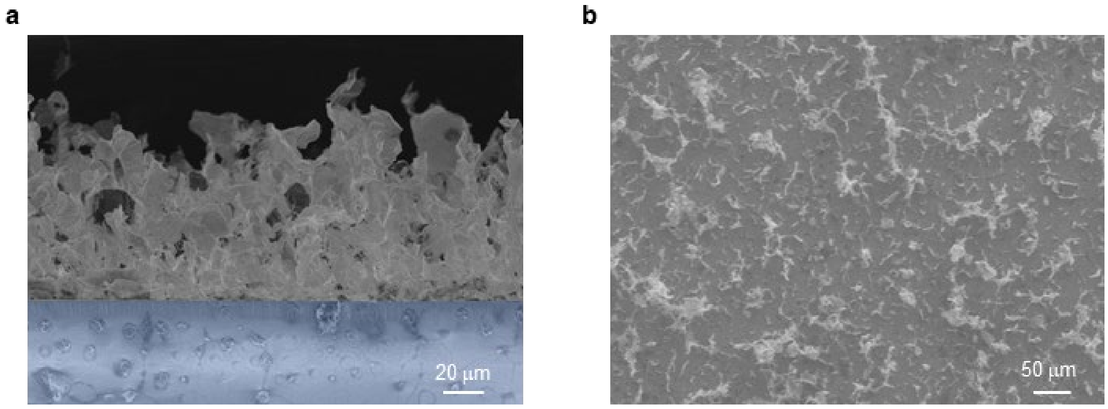

Supplementary Fig. 12 | SEM images of vertical reduced graphene oxide arrays in G/PEDOT, the top-view (a) and lateral-view (b) images.

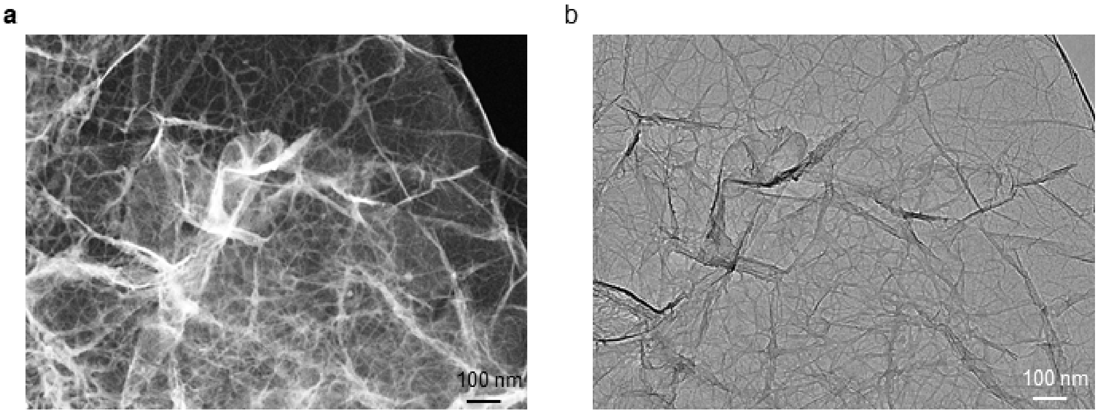

Supplementary Fig. 13 | TEM images of G/PEDOT, the HADDF mode (a) and transmission mode (c) images.

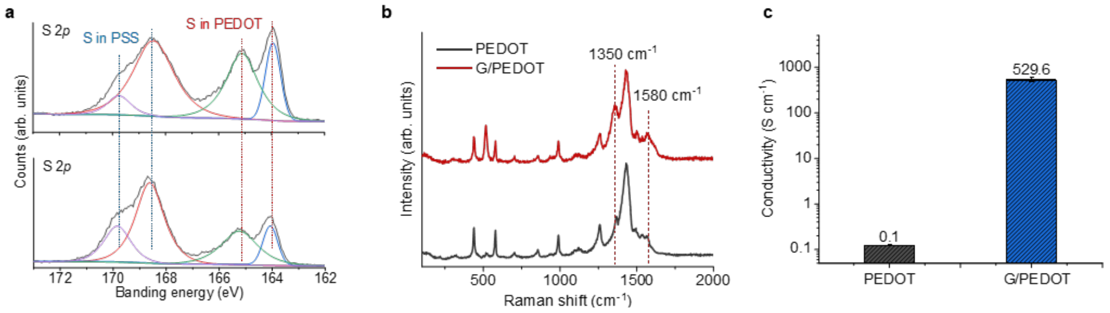

Supplementary Fig. 14 | Characterization of PEDOT:PSS and G/PEDOT. a, S 1*s* XPS spectra of PEDOT:PSS (upper panel) and G/PEDOT (bottom panel). **b**, Raman spectra of PEDOT:PSS and G/PEDOT. **c**, Electronic conductivity of PEDOT:PSS and G/PEDOT (n = 3, standard deviation).

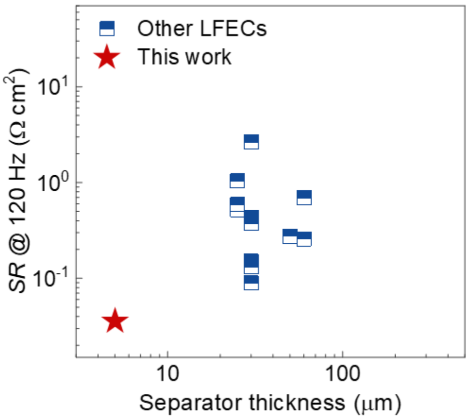

Supplementary Fig. 15 | Comparison of *SR* at 120 Hz and separator thickness between TAS-LFECs and other LFECs electrochemical reported previously.

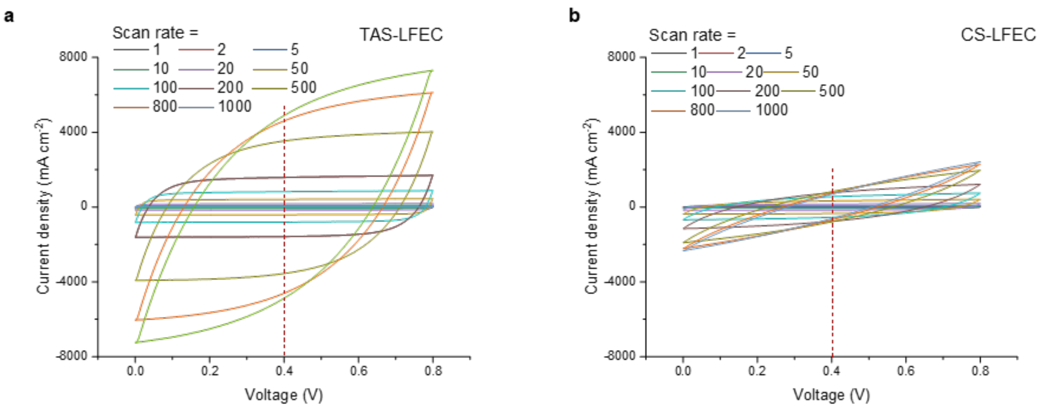

Supplementary Fig. 16 | Cyclic voltammetry plots of the TAS-LFEC (a) and CS-LFEC (b) at varying scan rates from 1 V s^{-1} to 1,000 V s^{-1}.

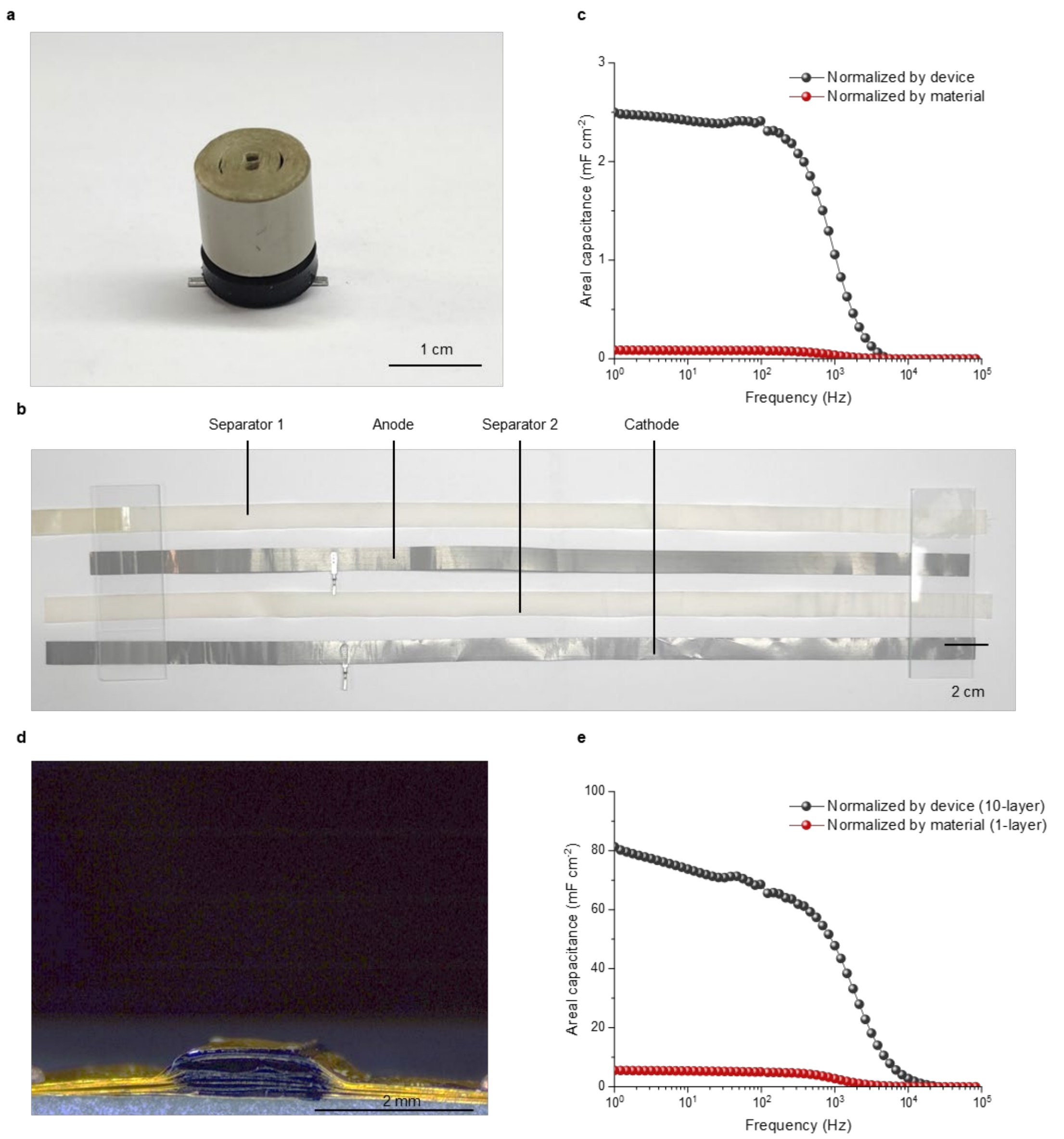

Supplementary Fig. 17 | Comparison of areal capacitance between TAS-LFEC and a commercial aluminum electrolytic capacitor. a,**b**, The optical images of a commercial aluminum electrolytic capacitor with a capacitance of 3.3 mF. **c**, Areal capacitance of the commercial aluminum electrolytic capacitor normalized by device or material. **d**, The optical images of the TAS-LFEC. **e**, Areal capacitance of TAS-LFECs normalized by device or material.

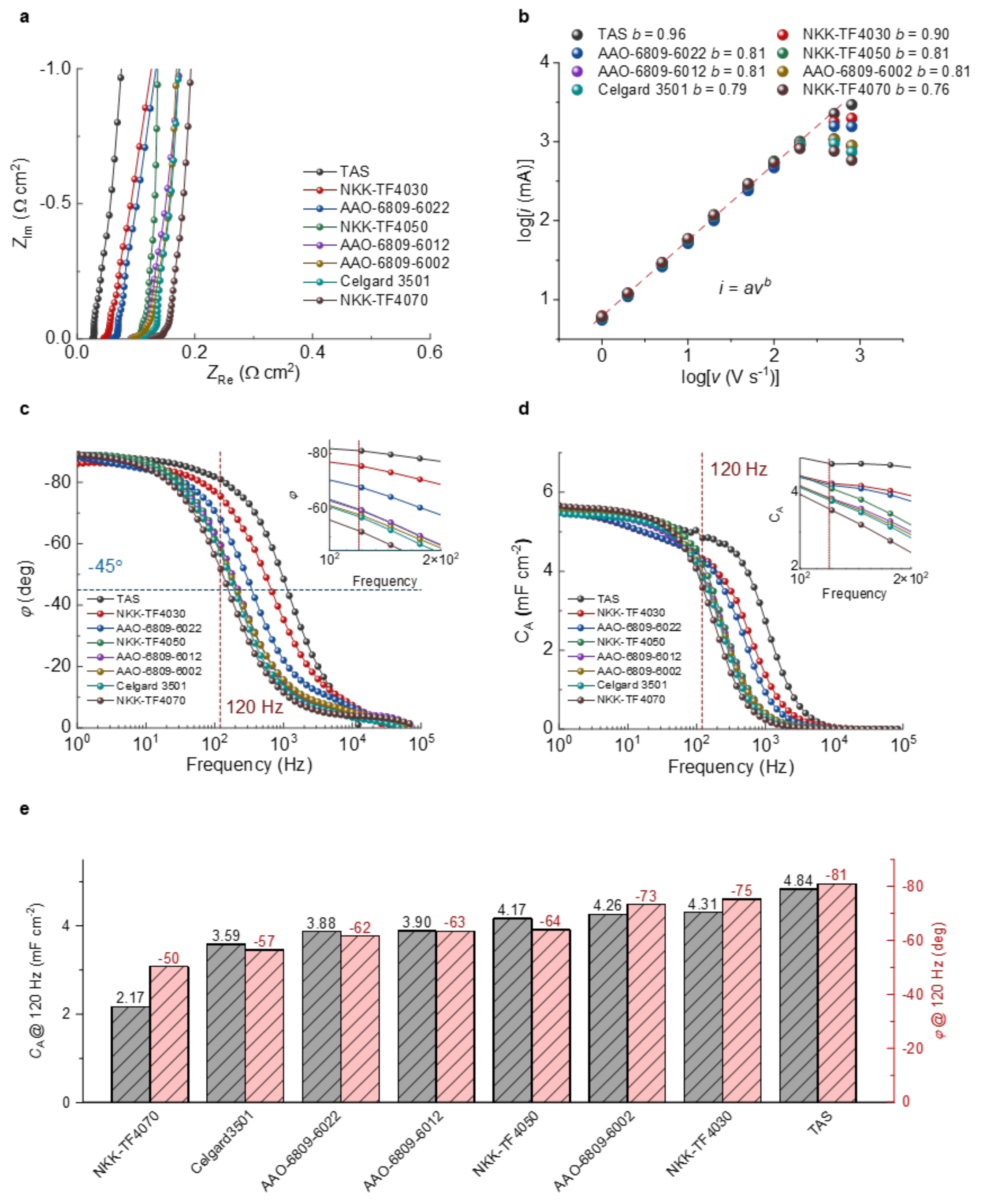

Supplementary Fig. 18 | Comparison of electrochemical properties between TAS-LFECs and electrochemical capacitors using commercial separators. a, Nyquist diagrams of TAS-LFECs and electrochemical capacitors using commercial separators. **b**, Plot of the logarithm of current density (i) versus the logarithm of scan rates (v) for TAS-LFECs and electrochemical capacitors using commercial separators. The red dashed line is the fitting curve, and the b value is determined from the slope of the curve.

c, Bode diagrams of TAS-LFECs and electrochemical capacitors using commercial separators. **d**, Plot of C_A with respect to frequency of TAS-LFECs and electrochemical capacitors using commercial separators. **e**, Comparison of C_A and φ at 120 Hz of TAS-LFECs and electrochemical capacitors using commercial separators.

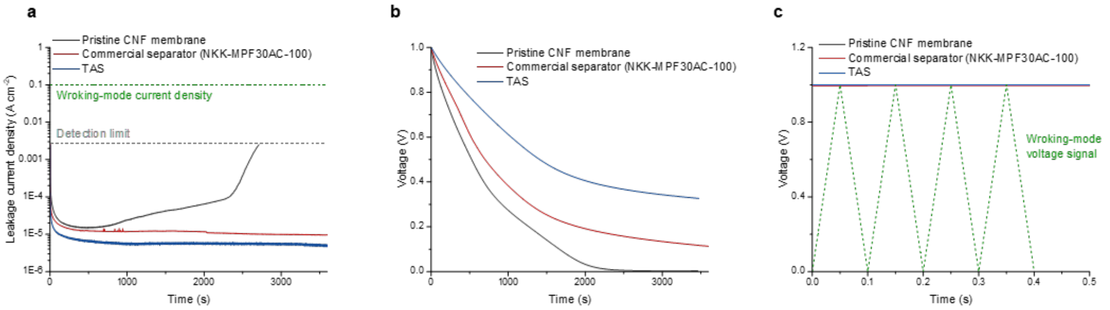

Supplementary Fig. 19 | Comparison of leakage current density (a) and self-discharge behavior (b,c) between Pristine CNF membrane, Commercial separator, and TAS.

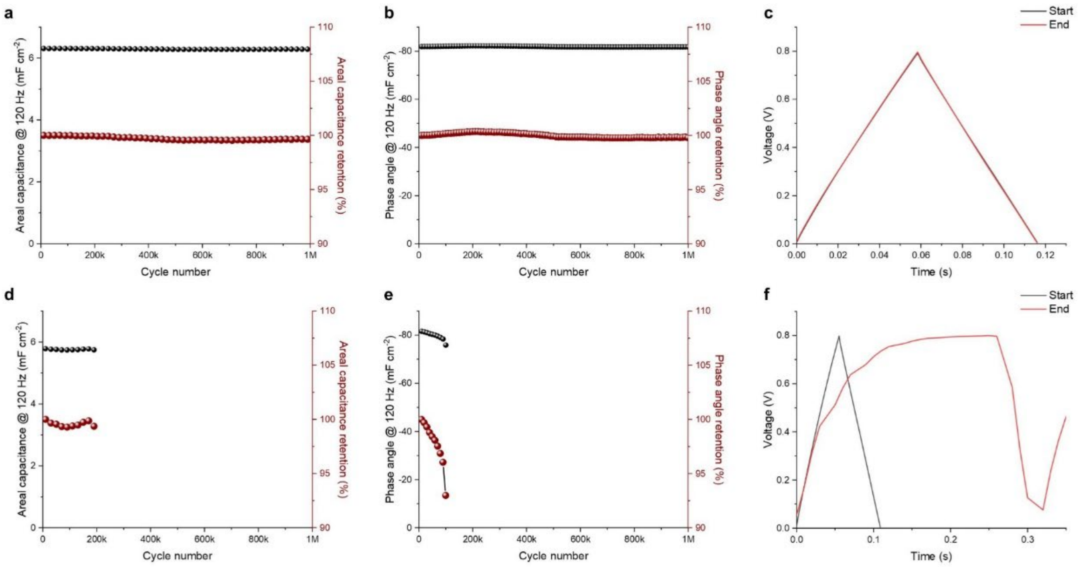

Supplementary Fig. 20 | Cyclic tests between TAS-LFEC and pristine CNF membrane-based LFEC. a, Areal capacitance at 120 Hz of TAS-LFEC during cycling test. **b**, Phase angle at 120 Hz of TAS-LFEC during cycling test. **c**, Galvanostatic curves of the TAS-LFEC at the start and end of the cycling test. **d**, Areal capacitance at 120 Hz of pristine CNF membrane-based LFEC during cycling test. **e**, Phase angle at 120 Hz of pristine CNF membrane-based LFEC during cycling test. **f**, Galvanostatic curves of the pristine CNF membrane-based LFEC at the start and end of the cycling test.

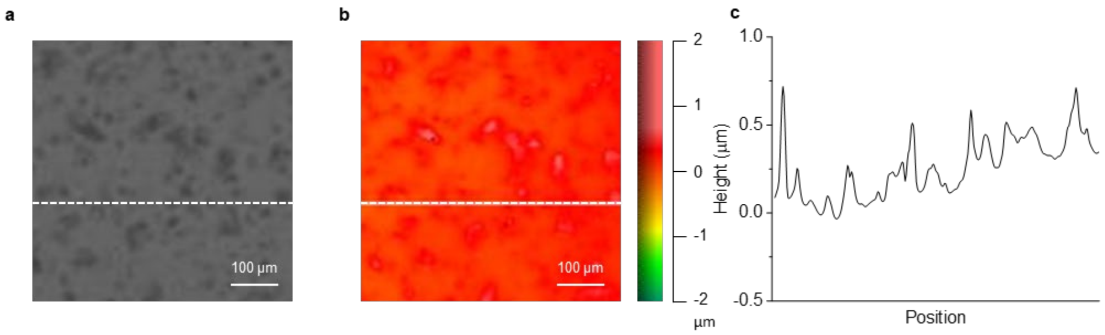

Supplementary Fig. 21 | Surface morphology of the electrode material G/PEDOT. **a**, Top-view optical image of the surface of G/PEDOT. **b**, 3-dimensional while light interferometry of G/PEDOT. **c**, Height information of the white dashed lines in **a** and **b**.

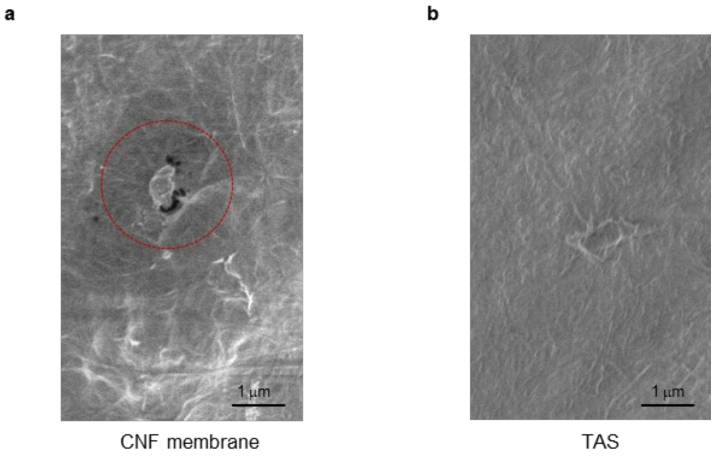

Supplementary Fig. 22 | Top-view SEM images of the separators after the test. a, The pristine CNF membrane. The red dashed circle indicates the protrusion induced by the assembly stress. **b**, The TAS.

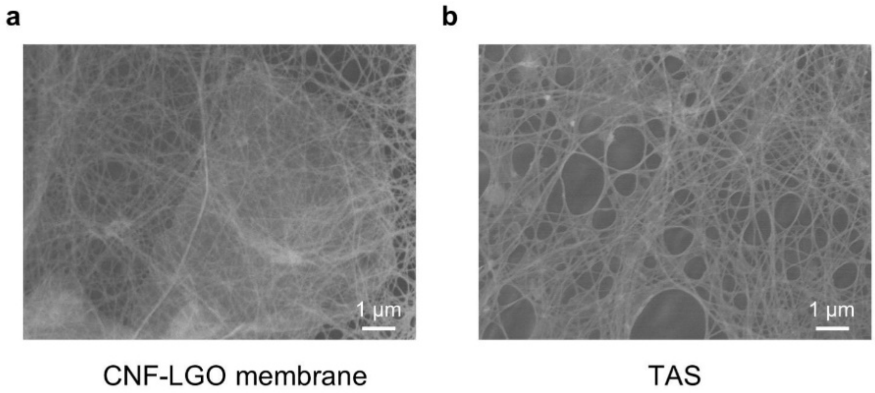

Supplementary Fig. 23| Top-view SEM images of the separators after the test. a, The CNF-LGO membrane showing the blocked pores by LGO. **b**, The TAS.

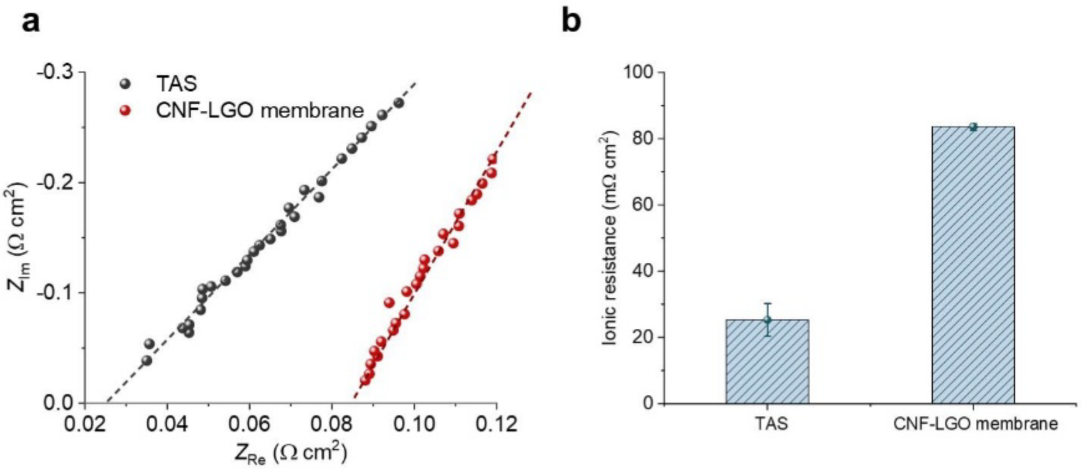

Supplementary Fig. 24 | Comparison of membrane ionic resistance between TAS and CNF-LGO membrane. a, Nyquist diagrams of TAS and CNF-LGO membrane. **b**, Ionic resistance of TAS and CNF-LGO membrane. The electrolyte is a 3 mol L^{-1} H_2SO_4 solution.

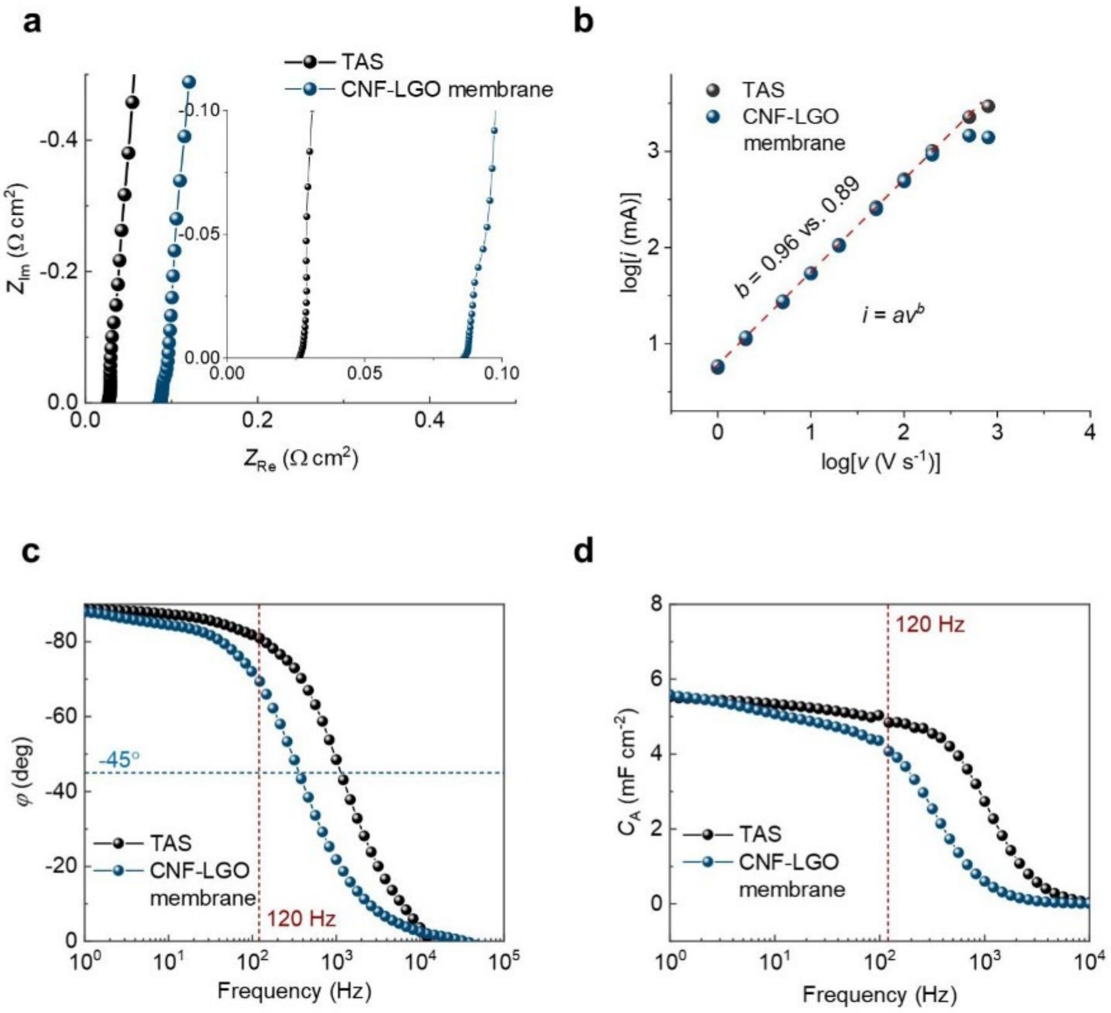

Supplementary Fig. 25 | Electrochemical performances of TAS-LFEC and the CNF-LGO-membrane-based-LFEC. a, Nyquist diagrams of TAS-LFECs and CNF-LGO-membrane-based-LFEC. **b**, The plot of the logarithm of current density (i) versus the logarithm of scan rates (v) for TAS-LFEC and CNF-LGO-membrane-based-LFEC. The red dashed line is the fitting curve, and the b value is determined from the slope of the curve. **c**, Bode diagrams of TAS-LFEC and CNF-LGO-membrane-based-LFEC. **d**, Plot of C_A with respect to frequency of TAS-LFEC and CNF-LGO-membrane-based-LFEC.

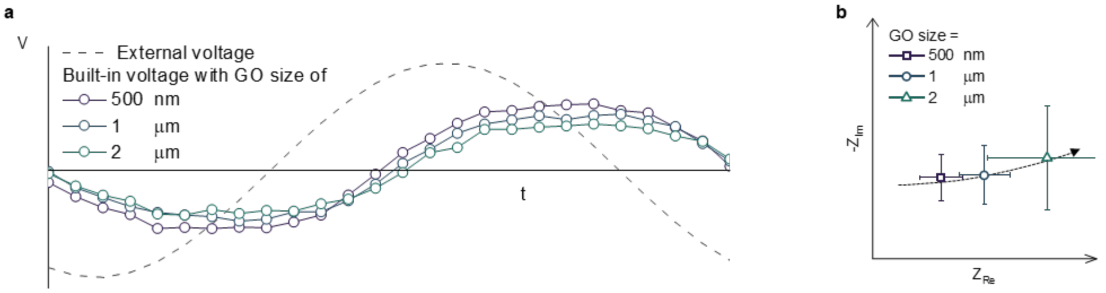

Supplementary Fig. 26 | Kinetic Monte Carlo simulation of the hysteresis behaviors of TAS-LFECs with different GO sheet sizes. **a**, Simulated hysteresis curves of TAS-LFECs with GO sheet sizes of 500 nm, 1 μm, and 2 μm. **b**, Simulated Nyquist impedances of TAS-LFECs with GO sheet sizes of 500 nm, 1 μm, and 2 μm.

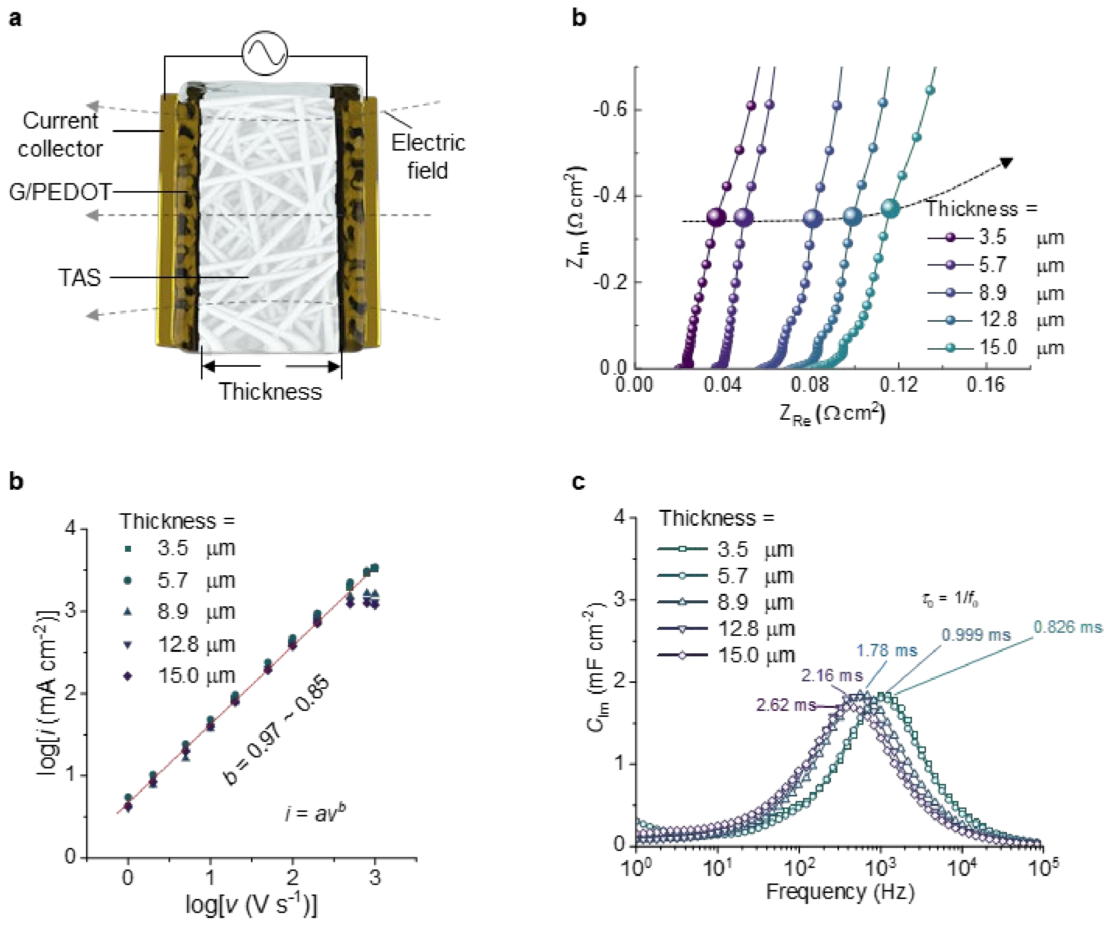

Supplementary Fig. 27 | Electrochemical performances of TAS-LFECs with varying thicknesses of TAS. a, Schematic diagram of TAS-LFECs with varying thicknesses of TAS. **b**, Plot of the logarithm of current density (*i*) versus the logarithm of scan rates (*v*) for TAS-LFECs with varying thicknesses of TAS. The red dashed line is the fitting curve, and the *b* value is determined from the slope of the curve. **c**, Plot of the imaginary part of capacitance with respect to frequency of TAS-LFECs with varying thicknesses of TAS.

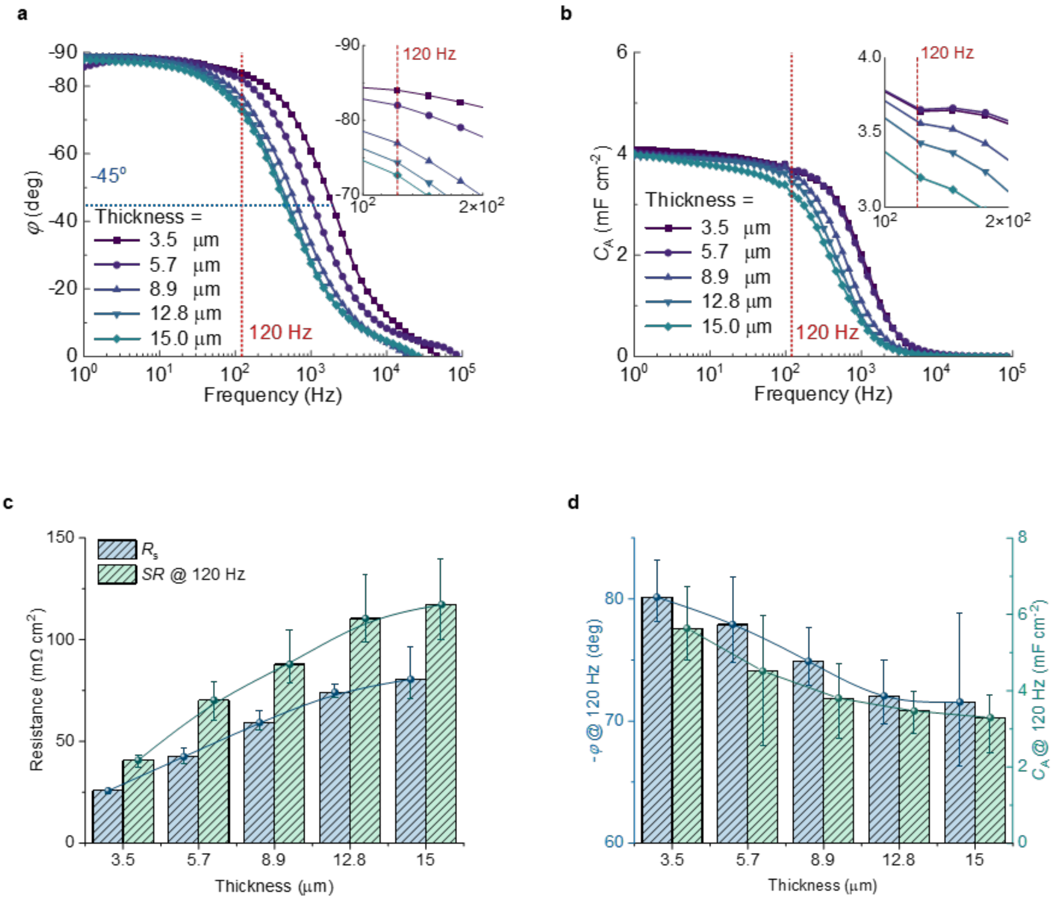

Supplementary Fig. 28 | Electrochemical performances of TAS-LFECs with varying thicknesses of TAS. a, Bode diagrams of TAS-LFECs with varying thicknesses of TAS. **b**, Plot of C_A with respect to frequency of TAS-LFECs with varying thicknesses of TAS. **c**, Histogram of R_s and *SR* at 120 Hz of TAS-LFECs with varying thicknesses of TAS (n = 3, standard deviation). **d**, Histogram of $-\varphi$ and C_A at 120 Hz of TAS-LFECs with varying thicknesses of TAS (n = 3, standard deviation).

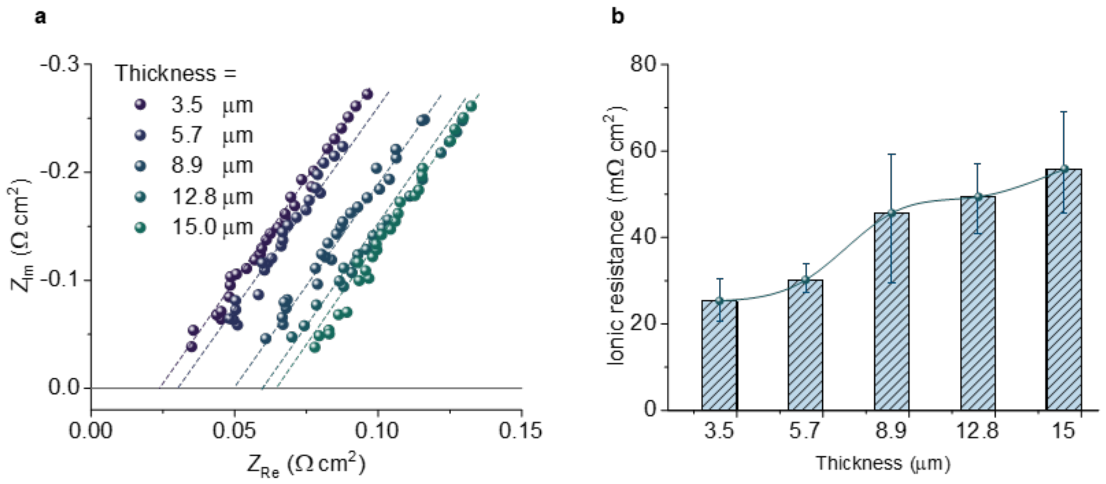

Supplementary Fig. 29 | Membrane ionic resistance of TAS with different thicknesses. a, Nyquist diagram of TAS with different thicknesses. **b**, Histogram of membrane ionic resistance of TAS with different thicknesses (n = 3, standard deviation). The electrolyte is a 3 mol L^{-1} H_2SO_4 solution.

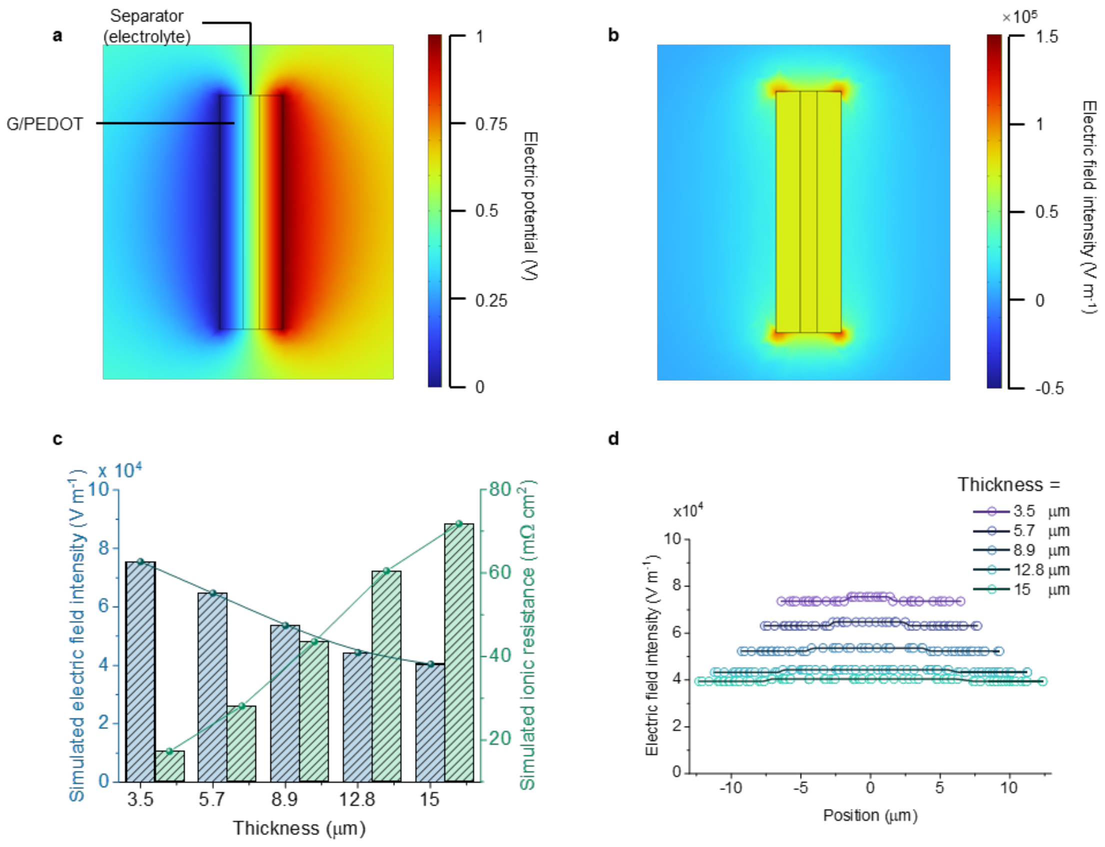

Supplementary Fig. 30 | Finite elemental analysis of TAS-LFECs with varying TAS thickness. a, Cross-sectional electric potential distribution of TAS-LFEC with 3.5-μm TAS. **b**, Cross-sectional electric field intensity of TAS-LFEC with 3.5-μm TAS. **c**, Histograms of simulated electric field intensity and simulated ionic resistance of TAS-LFECs with varying TAS thickness. **d**, Simulated electric field intensity distribution along the cross-section of TAS-LFECs with varying TAS thickness.

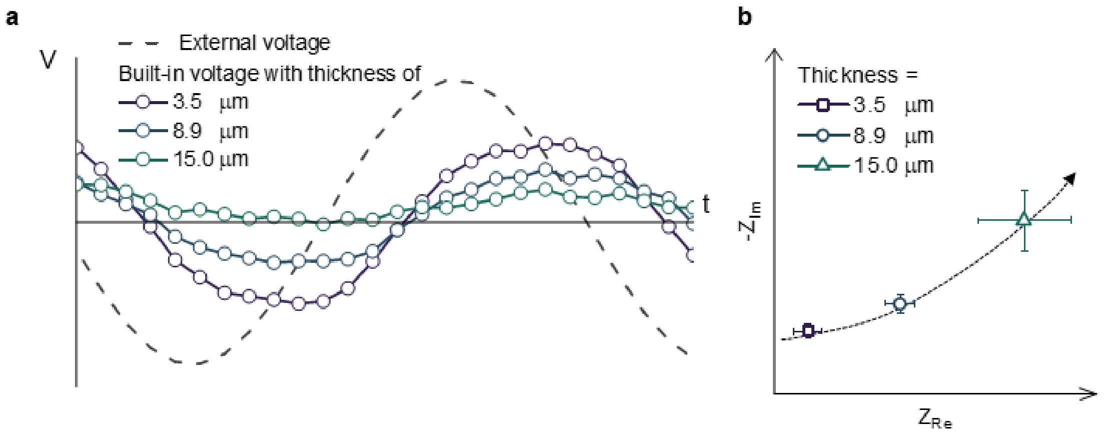

Supplementary Fig. 31 | Kinetic Monte Carlo simulation of the hysteresis behaviors of TAS-LFECs with different TAS thicknesses. a, Simulated hysteresis curves of TAS-LFECs with TAS thickness of 3.5 μm, 8.9 μm, and 15.0 μm. **b**, Simulated Nyquist impedances of TAS-LFECs with TAS thickness of 3.5 μm, 8.9 μm, and 15.0 μm.

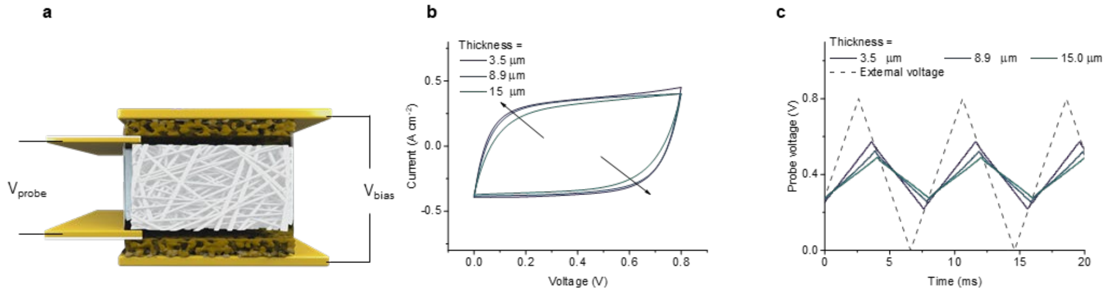

Supplementary Fig. 32 | Operando probing of ionic migration. a, Schematic diagram of the experimental apparatus. **b**, Cyclic voltammetry of TAS-LFECs with varying TAS thickness. The arrows indicate that polarization is lessened as the TAS thickness decreases. **c**, The probe voltage signals of TAS-LFECs with varying TAS thickness.

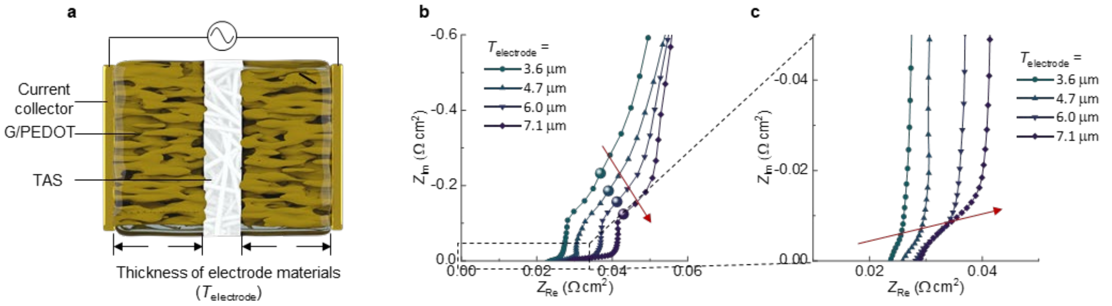

Supplementary Fig. 33 | Electrochemical performances of TAS-LFECs with varying electrode material thickness. a, Schematic diagram of TAS-LFECs with varying electrode material thickness. **b**,**c**, Nyquist diagrams of TAS-LFECs with varying electrode material thickness. Red arrows indicate the tendency of impedances as the electrode material thickness increases.

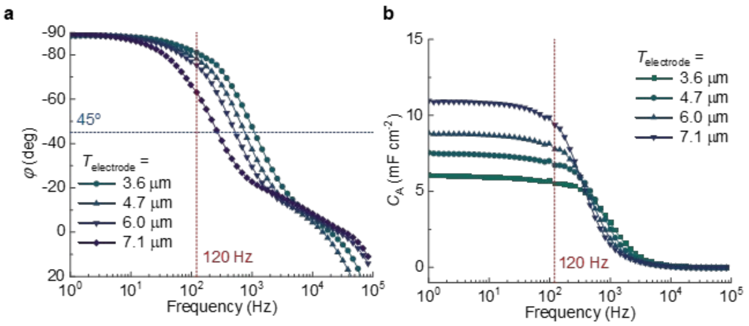

Supplementary Fig. 34 | Electrochemical performances of TAS-LFECs with varying electrode material thickness. **a**, Bode diagrams of TAS-LFECs with varying electrode material thickness. **b**, Plot of C_A with respect to frequency of TAS-LFECs with varying electrode material thickness.

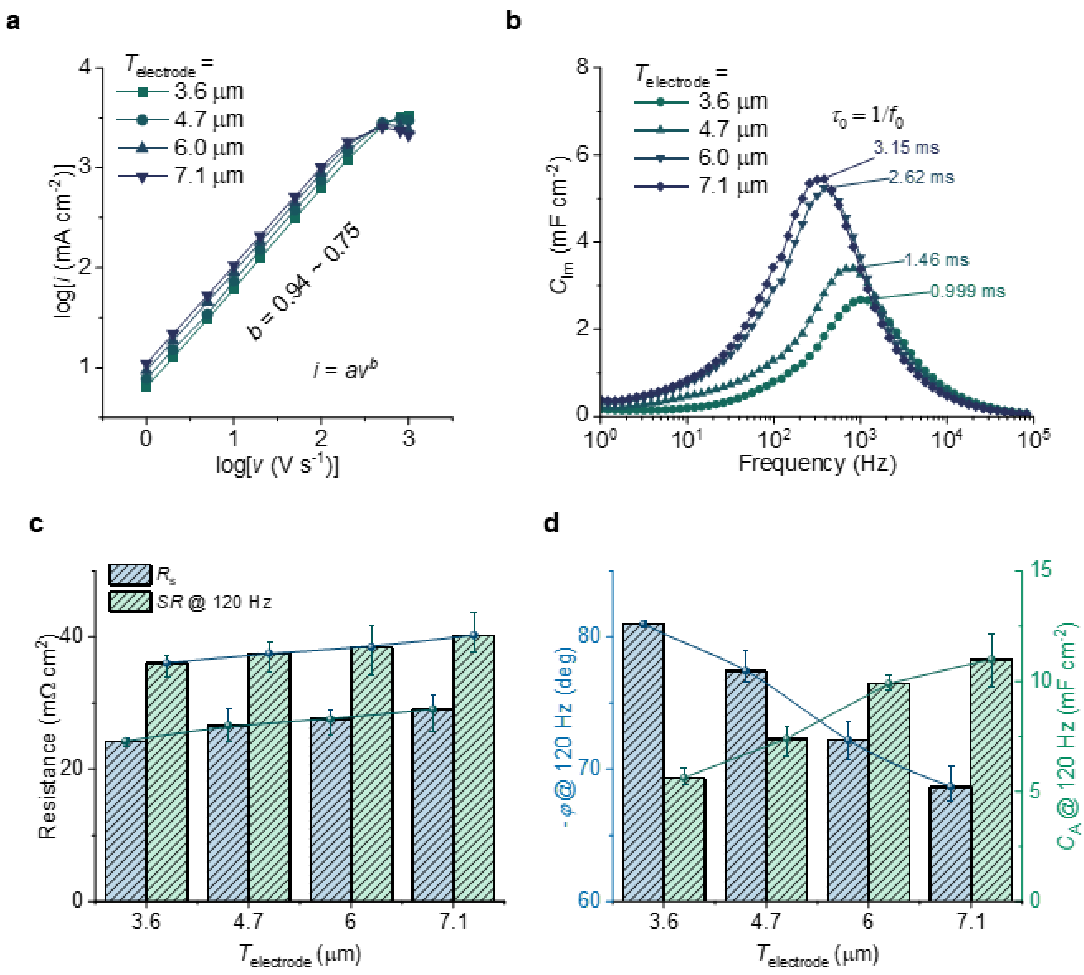

Supplementary Fig. 35 | Electrochemical performances of TAS-LFECs with varying electrode material thickness. a, Plot of the logarithm of current density (*i*) versus the logarithm of scan rates (*v*) for TAS-LFECs with varying electrode material thickness. **b**, Plot of the imaginary part of capacitance with respect to frequency of TAS-LFECs with varying electrode material thickness. τ_0 is the relaxation time constant, which reflects the typical response speed of the capacitor. f_0 is the frequency when C_{Im} reaches the maximum. **c**, Histogram of R_s and *SR* at 120 Hz of TAS-LFECs with varying electrode material thickness (n = 3, standard deviation). **d**, Histogram of $-\varphi$ at 120 Hz and C_A at 120 Hz of TAS-LFECs with varying electrode material thickness (n = 3, standard deviation).

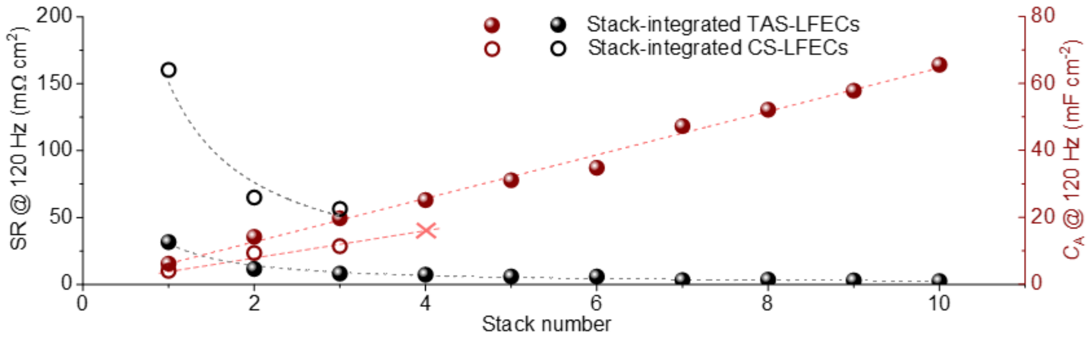

Supplementary Fig. 36 | Plots of *SR* and C_A at 120 Hz versus stack number of stack-integrated TAS-LFECs and CS-LFECs. The dashed lines are the fitting curves. The 'x' mark indicates the stack number at which the stack integration failed.

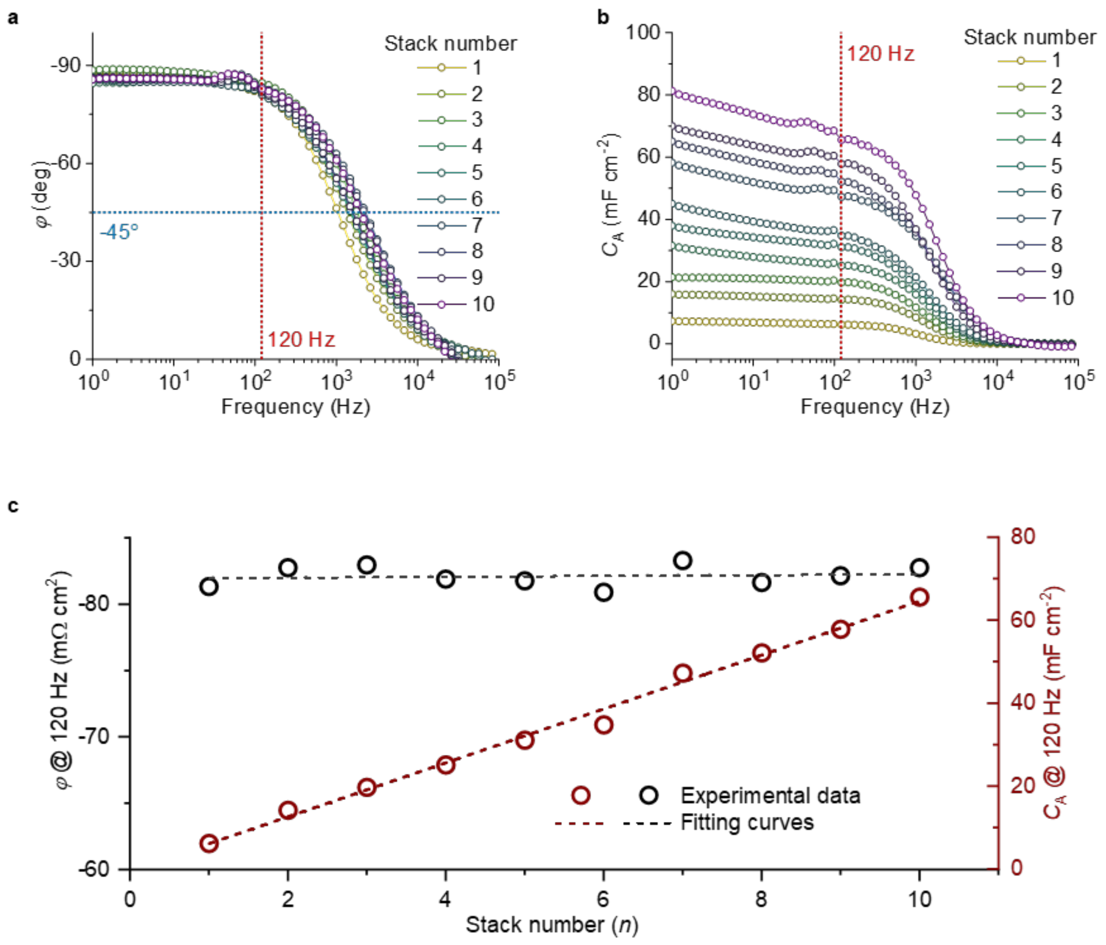

Supplementary Fig. 37 | Electrochemical performances of stack-integrated TAS-LFECs. a, Bode diagrams of stack-integrated TAS-LFECs with different stack numbers. **b**, Plot of C_A with respect to frequency of stack-integrated TAS-LFECs with different stack numbers. **c**, The plot of φ at 120 Hz and C_A at 120 Hz versus stack number of stack-integrated TAS-LFECs. The dashed lines are the fitting curves.

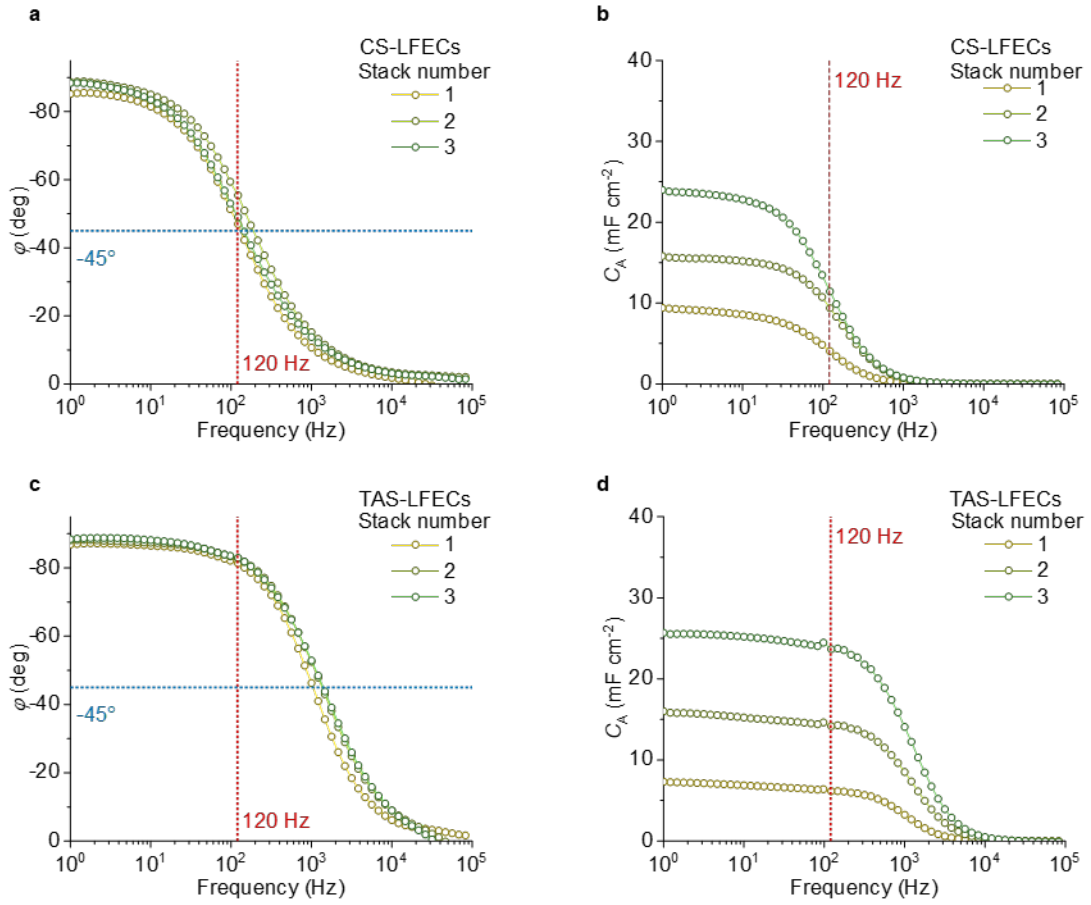

Supplementary Fig. 38 | Comparison of electrochemical performances between the stack-integrated TAS-LFECs and CS-LFECs with stack numbers varying from 1-3. **a**, Bode diagrams of the stack-integrated CS-LFECs with stack numbers varying from 1-3. **b**, Plots of areal capacitance versus frequency of the stack-integrated CS-LFECs with stack numbers varying from 1-3. **c**, Bode diagrams of the stack-integrated TAS-LFECs with stack numbers varying from 1-3. **d**, Plots of areal capacitance versus frequency of the stack-integrated TAS-LFECs with stack numbers varying from 1-3.

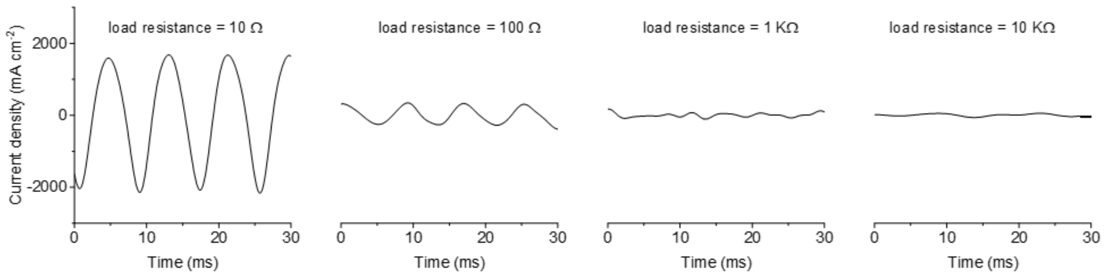

Supplementary Fig. 39 | Ripple current of a 10-layer stack-integrated TAS-LFEC with resistance varying from 10 Ω to 10,000 Ω.

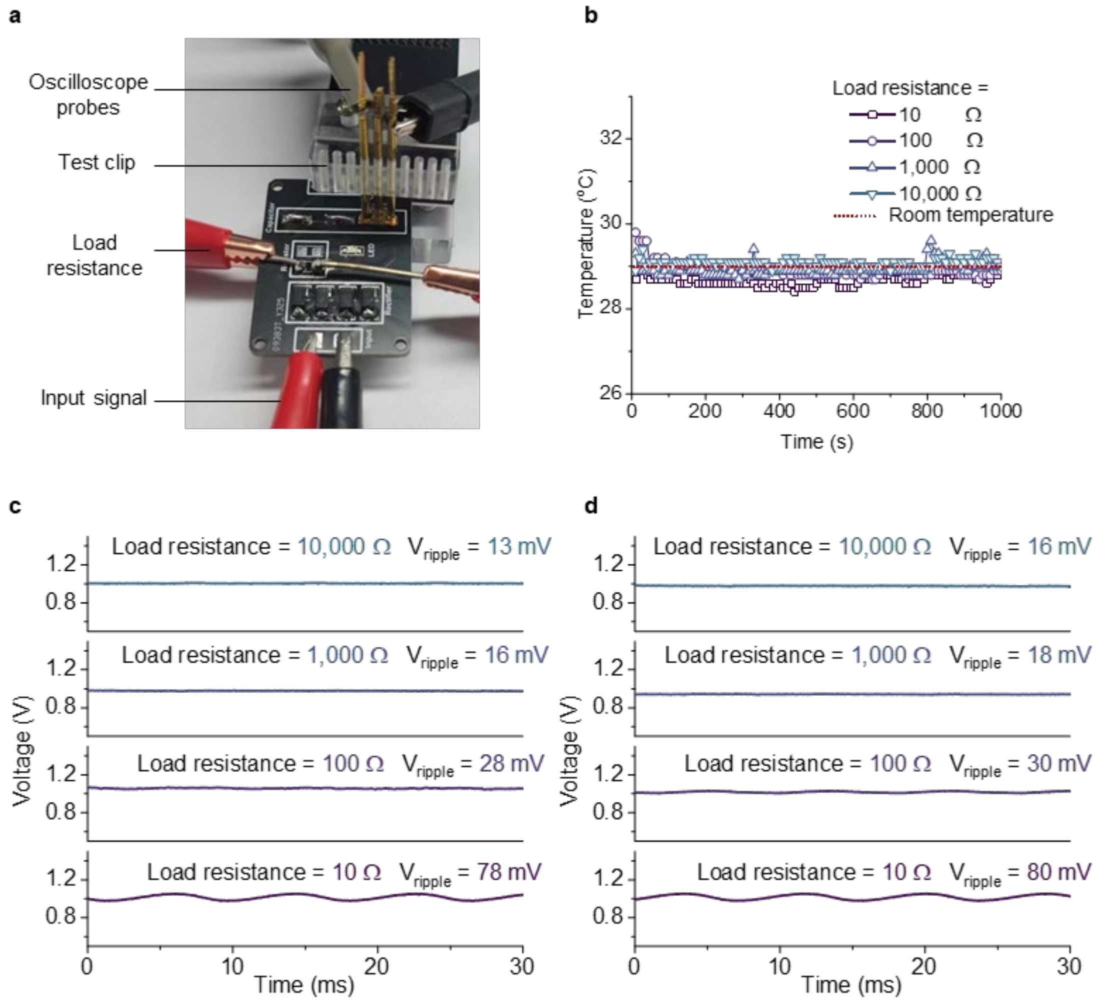

Supplementary Fig. 40 | Temperature tracing of a 10-layer stack-integrated TAS-LFEC with resistance varying from 10 Ω to 10,000 Ω. a, Optical image of the test apparatus. **b**, Temperature variation of a 10-layer stack-integrated TAS-LFEC with resistance varying from 10 Ω to 10,000 Ω. **c**, Initial oscilloscopic waveforms of the output signal of a 10-layer stack-integrated TAS-LFEC with resistance varying from 10 Ω to 10,000 Ω. **d**, After 2 hours' oscilloscopic waveforms of the output signal of a 10-layer stack-integrated TAS-LFEC with resistance varying from 10 Ω to 10,000 Ω.

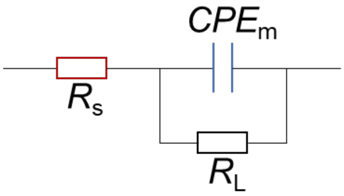

Supplementary Fig. 41 | Equivalent circuit of a general line-filtering electrochemical capacitor. R_s refers to the ionic resistance within separators, CPE_m represents the unideal capacitance contributed by the electrode materials, and R_L indicates the faradaic resistance, which contributes to the leakage current of the ELFC.

Supplementary Table 1 | Comparison of line-filtering performances between a 10-layer stack-integrated TAS-LFEC with other electrochemical line-filtering capacitors at varying load power densities.

Electrode material	Separator	φ@ 120Hz (deg)	C_A@ 120Hz (mF cm^{-2})	Area (cm^2)	Current density(mA cm^{-2})	Load power density(mW cm^{-2})	Resistance (Ω)	Ripple voltage (mV)	Input voltage (V)	Ripple factor (%)	Reference
**This work (10-layer stack-integrated TAS-LFEC)**	**This work (10-layer stack-integrated TAS-LFEC)**	**-83**	**47**	**0.04**	**2,500**	**2,500**	**10**	**73**	**1.5**	**4.9**	-
					**250**	**250**	**100**	**27**	**1.4**	**1.9**	-
					**25**	**25**	**1,000**	**19**	**1.2**	**1.6**	-
					**2.5**	**2.5**	**10,000**	**16**	**1.2**	**1.3**	-
Prussian blue based vertical graphene	Celgard	-80.6	2.34	1.91	3.35	10.05	470	40	3	18	[1]
Ketjen black	PTFE	-80	0.574	17.3	0.22	1.848	2,200	-	8.4	-	[2]
Carbon+carbon nanotubes	NKK-TF4030	-80.5	2.81	1.54	0.39	2.34	10000	7.09	6	0.14	[3]
Reduced graphene oxide+PEDOT	AAO membrane	-82	0.27	0.25	0.8	1.6	10000	20	2	1.3	[4]
Vertical graphene	AAO membrane	-80.6	1.72	0.25	0.004	0.004	1000000	-	1	-	[5]
Carbon nanotubes	PTFE	-78.1	0.106	1	0.3	0.9	10000	500	3	16.7	[6]
Reduced graphene oxide fiber	PVA/H_2SO_4	-81.1	0.264	-	-	<0.001	no load	50	1	5	[7]
TiN_x	-	-65	1.6	0.32	-	<0.001	no load	30	1.8	1.6	[8]
MXene	cellulose membrane	-80	1.14	0.25	-	<0.001	no load	42	1.4	3	[9]
Mxene+PEDOT	polymeric hydrogel electrolyte	-79.1	0.56	36	-	<0.001	no load	125	1.5	8.3	[10]
Reduced graphene oxide+PEDOT	cellulose membrane	-81	0.451	1	2.5	0.625	100	10	0.25	4	[11]
Graphitic ordered mesoporous carbon	PTFE	-80	0.559	17.3	0.12	0.24	1000	228	2	11.4	[12]
Carbon nanotubes+PEDOT	NKK-TF4050	-83.3	0.573	0.25	0.0008	0.0016	10,000,000	10	2	0.5	[13]
Reduced graphene oxide	AAO membrane	-80	0.149	0.25	-	<0.001	no load	5.6	3	0.18	[14]
PEDOT:PSS/Ketjenblack holey nanosheets	NKK-TF4050	-81.9	3.09	0.36	0.0028	0.028	10,000,000	233	10	2.33	[15]

Supplementary Table 2 | Statistics of thicknesses and ionic resistances of electrode materials and separators.

Electrode material	Separator	Thickness of electrode material (μm)	Thickness of separator (μm)	R_s (Ω cm^2)	R_m @ 120 Hz (Ω cm^2)	R_s/R_s+R_m (%)	SR @ 120 Hz (Ω cm^2)	Reference
Vertical graphene	Microporous separator	0.6	25	1.00	0.04	96	1.04	[16]
Graphene	Celgard 3501	2	25	0.22	0.30	42	0.52	[17]
Ultrathin free-standing graphene nanomesh	Celgard 3501	0.45	25	0.39	0.20	66	0.59	[18]
Carbon nanotube	PTFE membrane	0.13	30	1.20	1.45	45	2.65	[6]
Nitrogen doped holey graphene	PTFE membrane	2	30	0.20	0.23	46	0.43	[19]
PEDOT	NKK-TF4030	0.6	30	0.09	0.06	60	0.15	[20]
Fabric carbon	NKK-TF4030	5	30	0.05	0.04	56	0.09	[21]
Reduced graphene oxide and PEDOT	NKK-TF4030	0.12	30	0.15	0.22	40	0.37	[22]
Carbon nanotube and PEDOT	NKK-TF4050	0.2	50	0.07	0.20	26	0.27	[13]
PEDOT:PSS/Ketjenblack holey nanosheets	NKK-TF4050	1.5	50	0.05	0.01	83	0.06	[15]
Reduced graphene oxide and PEDOT	AAO membrane	20	60	0.21	0.48	30	0.69	[4]
Vertical graphene	AAO membrane	5	60	0.13	0.13	50	0.26	[5]
Carbon and Carbon nanotube	MPF30AC-100	10	100	0.11	0.03	78	0.13	[3]
Average		-	-	-	-	54	-	-

Supplementary Table 3 | Comparison of φ at 120 Hz and C_A at 120 Hz between TAS-LFECs and other electrochemical line-filtering capacitors.

Electrode material	Separator	Thickness (μm)	φ @ 120Hz (deg)	C_A @ 120Hz (mF cm^{-2})	Reference
This work (G/PEDOT)	This work (TAS)	3	-80.0	6.6	-
		3	-81.2	5.3	-
		3	-82.2	4.4	-
		3	-83.2	4.0	-
		3	-85.2	2.7	-
Carbon tubes	MPF-30AC-100	100	-80	2.81	[3]
Carbon tubes	MPF-30AC-100	100	-80.1	3.08	[23]
Fabric carbon	NKK-TF4030	30	-80	2.632	[21]
Mxene	NKK-TF4030	30	-80	1.14	[9]
PEDOT	NKK-TF4030	30	-83.6	0.994	[20]
Reduced graphene oxide+PEDOT	NKK-TF4030	30	-81.4	0.543	[22]
Prussian blue based vertical graphene	Celgard 3501	25	-80.6	2.34	[1]
Graphene	Celgard 3501	25	-82	0.36	[17]
Vertical graphene	AAO membrane	60	-80.6	1.72	[5]
Electroreduced graphene oxide	AAO membrane	60	-80.5	0.472	[24]
Electroreduced graphene oxide+PEDOT	AAO membrane	60	-82	0.27	[4]
Ketjen black	PTFE membrane	30	-80	0.574	[2]
Graphitic ordered mesoporous carbon	PTFE membrane	30	-80	0.559	[12]

Supplementary References

1 Li, W., Azam, S., Dai, G. & Fan, Z. Prussian blue based vertical graphene 3D structures for high frequency electrochemical capacitors. *Energy Stor. Mater.* **32**, 30-36 (2020).

2 Yoo, Y., Park, J., Kim, M.-S. & Kim, W. Development of 2.8 V Ketjen black supercapacitors with high rate capabilities for AC line filtering. *J. Power Sources* **360**, 383-390 (2017).

3 Han, F. *et al.* Structurally integrated 3D carbon tube grid-based high-performance filter capacitor. *Science* **377**, 1004-1007 (2022).

4 Wu, M. *et al.* Arbitrary waveform AC line filtering applicable to hundreds of volts based on aqueous electrochemical capacitors. *Nat. Commun.* **10**, 2855 (2019).

5 Xu, S. *et al.* Vertical graphene arrays as electrodes for ultra-high energy density AC line-filtering capacitors. *Angew. Chem. Int. Ed.* **60**, 24505-24509 (2021).

6 Kang, Y. J., Yoo, Y. & Kim, W. 3-V Solid-state flexible supercapacitors with ionic-liquid-based polymer gel electrolyte for AC line filtering. *ACS Appl. Mater. Interfaces* **8**, 13909-13917 (2016).

7 Zhao, J. *et al.* Fiber-shaped electrochemical capacitors based on plasma-engraved graphene fibers with oxygen vacancies for alternating current line filtering performance. *ACS Appl. Energy Mater.* **2**, 993-999 (2019).

8 Wang, F. *et al.* Laser-iInduced transient self-organization of TiN(x) nano-filament percolated networks for high performance surface-mountable filter

capacitors. *Adv. Mater.* **35**, e2210038 (2023).

9 Wen, Y., Chen, H., Wu, M. & Li, C. Vertically oriented MXene bridging the frequency response and capacity density gap for AC-filtering pseudocapacitors. *Adv. Funct. Mater.* **32**, 2111613 (2022).

10 Gund, G. S. *et al.* MXene/polymer hybrid materials for flexible AC-filtering electrochemical capacitors. *Joule* **3**, 164-176 (2019).

11 Wang, L. *et al.* Alternatingly stacked thin film electrodes-based compact aqueous hybrid electrochemical capacitors for hundred-volts AC line filtering. *J. Energy Chem.* **78**, 158-168 (2023).

12 Yoo, Y., Kim, M.-S., Kim, J.-K., Kim, Y. S. & Kim, W. Fast-response supercapacitors with graphitic ordered mesoporous carbons and carbon nanotubes for AC line filtering. *J. Mater. Chem. A* **4**, 5062-5068 (2016).

13 Li, Z. *et al.* Aqueous hybrid electrochemical capacitors with ultra-high energy density approaching for thousand-volts alternating current line filtering. *Nat. Commun.* **13**, 6359 (2022).

14 Chi, F. *et al.* Graphene ionogel ultra-fast filter supercapacitor with 4 V workable window and 150 degrees C operable temperature. *Small* **18**, 2200916 (2022).

15 Zhao, M. *et al.* PEDOT:PSS/Ketjenblack holey nanosheets with ultrahigh areal capacitance for kHz AC line-filtering micro-supercapacitors. *Adv. Funct. Mater.* **33**, 2313495 (2023).

16 Miller, J. R., Outlaw, R. A. & Holloway, B. C. Graphene double-layer capacitor with ac line-filtering performance. *Science* **329**, 1637-1639 (2010).

17 Ren, G., Pan, X., Bayne, S. & Fan, Z. Kilohertz ultrafast electrochemical supercapacitors based on perpendicularly-oriented graphene grown inside of nickel foam. *Carbon* **71**, 94-101 (2014).

18 Zhang, Z. *et al.* Scalable fabrication of ultrathin free-standing graphene nanomesh films for flexible ultrafast electrochemical capacitors with AC line-filtering performance. *Nano Energy* **50**, 182-191 (2018).

19 Zhou, Q., Zhang, M., Chen, J., Hong, J. D. & Shi, G. Nitrogen-doped holey graphene film-based ultrafast electrochemical capacitors. *ACS Appl. Mater. Interfaces* **8**, 20741-20747 (2016).

20 Zhang, M. *et al.* An ultrahigh-rate electrochemical capacitor based on solution-processed highly conductive PEDOT:PSS films for AC line-filtering. *Energy Environ. Sci.* **9**, 2005-2010 (2016).

21 Zhang, M. *et al.* Bridged carbon fabric membrane with boosted performance in AC line-filtering capacitors. *Adv. Sci.* **9**, e2105072 (2022).

22 Zhang, M. *et al.* Robust graphene composite films for multifunctional electrochemical capacitors with an ultrawide range of areal mass loading toward high-rate frequency response and ultrahigh specific capacitance. *Energy Environ. Sci.* **11**, 559-565 (2018).

23 Chen, G. *et al.* Three-dimensional multi-layer carbon tube electrodes for AC line-filtering capacitors. *Joule* **8**, 1080-1091 (2024).

24 Chi, F. *et al.* Graphene-based organic electrochemical capacitors for AC line filtering. *Adv. Energy Mater.* **7**, 1700591 (2017).
