# Supplementary material for: Separator with high ionic conductivity enables electrochemical capacitors to line-filter at high power
Source: Nat Commun. 2025 Mar 20;16:2772. doi: 10.1038/s41467-025-58064-2 (PMC11926240; doi:10.1038/s41467-025-58064-2)
Supplement: Supplementary file 2 — Description of Additional Supplementary Files [file 41467_2025_58064_MOESM2_ESM.docx]

Description of Additional Supplementary Files

**File Name:** Supplementary Movie 1

**Description:** Kinetic Monte Carlo simulation of the built-in voltage of TAS-LFECs with different GO sheet sizes.

**File Name:** Supplementary Movie 2

**Description:** High-power line filtering test (Comparison between stackintegrated TAS-LFECs and the same-volume aluminum electrolytic capacitor)

**File Name:** Supplementary Data 1

**Description:** Molecular structure of the CNF-GO complex.
